# Calpain-mediated cleavage of collapsin response mediator protein-2 drives acute axonal degeneration

**DOI:** 10.1038/srep37050

**Published:** 2016-11-15

**Authors:** Jian-Nan Zhang, Uwe Michel, Christof Lenz, Caroline C. Friedel, Sarah Köster, Zara d’Hedouville, Lars Tönges, Henning Urlaub, Mathias Bähr, Paul Lingor, Jan C. Koch

**Affiliations:** 1 Department of Neurology, University Medicine Göttingen, 37075 Göttingen, Germany; 2 Bioanalytical Mass Spectrometry, Max Planck Institute for Biophysical Chemistry, 37077 Göttingen, Germany; 3 Institute of Clinical Chemistry, University Medicine Göttingen, 37075 Göttingen, Germany; 4 Institute for Informatics, LMU Munich, 80333 Munich, Germany; 5 Institute for X-Ray Physics, Georg-August-University Göttingen, 37077 Göttingen, Germany; 6 Center for Nanoscale Microscopy and Molecular Physiology of the Brain (CNMPB), 37075 Göttingen, Germany; 7 Department of Neurology, Ruhr-University Bochum, St. Josef-Hospital, 44791 Bochum, Germany

## Abstract

Axonal degeneration is a key initiating event in many neurological diseases. Focal lesions to axons result in a rapid disintegration of the perilesional axon by acute axonal degeneration (AAD) within several hours. However, the underlying molecular mechanisms of AAD are only incompletely understood. Here, we studied AAD *in vivo* through live-imaging of the rat optic nerve and *in vitro* in primary rat cortical neurons in microfluidic chambers. We found that calpain is activated early during AAD of the optic nerve and that calpain inhibition completely inhibits axonal fragmentation on the proximal side of the crush while it attenuates AAD on the distal side. A screening of calpain targets revealed that collapsin response mediator protein-2 (CRMP2) is a main downstream target of calpain activation in AAD. CRMP2-overexpression delayed bulb formation and rescued impairment of axonal mitochondrial transport after axotomy *in vitro. In vivo*, CRMP2-overexpression effectively protected the proximal axon from fragmentation within 6 hours after crush. Finally, a proteomic analysis of the optic nerve was performed at 6 hours after crush, which identified further proteins regulated during AAD, including several interactors of CRMP2. These findings reveal CRMP2 as an important mediator of AAD and define it as a putative therapeutic target.

Axonal degeneration is a prominent pathological feature in many neurological diseases, often occurring early during disease course and resulting in irreversible defects in neuronal connectivity^1^,^2^. In neurodegenerative diseases like Parkinson’s disease and Amyotrophic lateral sclerosis, axonal degeneration starts at the synaptic terminals and progresses slowly in a “dying back” manner towards the soma^3^,^4^,^5^. In chronic inflammatory diseases like multiple sclerosis, focal lesions of the axon result in axonal degeneration^6^. Traumatic lesions of axonal tracts like the spinal cord and the optic nerve lead to axonal degeneration in a well-defined spatial and temporal order^2^, employing intracellular signaling cascades, which can be compared to programmed cell death during apoptosis. Because of its reproducibility and convenient experimental accessibility, traumatic axonal degeneration has been a frequent subject of research^7^,^8^.

A focal traumatic lesion of an axon in the CNS is followed by a rapid fragmentation of the adjacent axon parts spanning up to 500 μm from the lesion within several hours. This process is termed acute axonal degeneration (AAD)^9^,^10^. After 24 to 72 hours, only the distal part of the axon undergoes a fragmentation called Wallerian degeneration (WD)^11^,^12^. The proximal part of the axon either slowly degenerates in a “dying-back” manner or remains stable. Lesioned axons have the potential to regenerate but inhibitory molecular cues from glial cells, scar tissue and a low intrinsic regenerative ability largely prevent successful axonal regeneration in the CNS^13^,^14^,^15^. The basis for the development of pro-regenerative treatments is a stabilization of the remaining axon requiring a better understanding of the molecular mechanisms of axonal degeneration, especially on the proximal side.

While a lot of progress has been made in understanding the molecular events underlying WD^12^, much less is known about AAD. It was shown in both spinal cord and optic nerve, that AAD is initiated by a transient rise in intracellular calcium concentrations^9^,^10^. In the spinal cord, a subsequent activation of the calcium-dependent protease calpain was reported^9^. Very soon after the elevation of calcium levels, an activation of autophagy is observed in the optic nerve followed by rapid cytoskeletal and mitochondrial disintegration resulting in the formation of axonal degeneration bulbs^10^. The underlying molecular mechanisms, however, are not fully understood.

The optic nerve is an excellent model structure to study axonal degeneration due to its convenient surgical accessibility, well-defined anatomy and the possibility to manipulate retinal ganglion cells (RGCs) by intravitreal injection of viral vectors or pharmacological substances. We have established a live-imaging set-up that allows for imaging of AAD in the anaesthetized living rat *in vivo*^16^. Axons are labeled by fluorophores expressed by adeno-associated viral vectors (AAV) that are injected into the vitreous space two weeks before a crush lesion of the optic nerve (ONC). Besides its role as an excellent model system for CNS axons, axonal degeneration of the optic nerve is involved in several clinically relevant human disorders, including optic neuritis, glaucoma and hereditary optic atrophy^17^.

In this study, we analyze the molecular mechanisms of AAD following the initial rise in intraaxonal calcium. We evaluate the role of calpain in AAD of the optic nerve and identify collapsin response mediator protein-2 (CRMP2) (also known as Dihydropyrimidinase-related protein 2 (DPYSL2)) as a major downstream target of calpain regulating AAD *in vitro* and *in vivo*.

## Results

### Calpain is activated early during acute axonal degeneration

**Figure 1:**
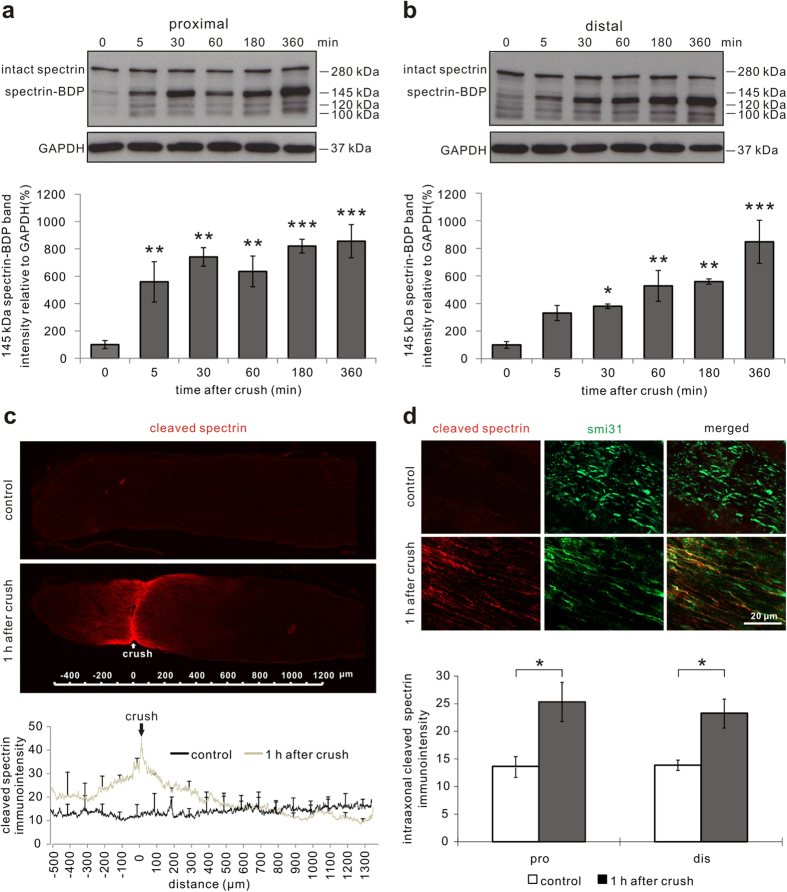
Calpain-mediated spectrin proteolysis during acute axonal degeneration after optic nerve crush *in vivo*. (**a,b**) Representative Western blots of spectrin proximal (**a**) and distal (**b**) to the crush site in the optic nerves at indicated time points after crush and uncrushed optic nerves (0 min after crush). Below, the quantification of the 145 kDa cleaved spectrin band is depicted, which represents the breakdown product (BDP) of spectrin specifically attributed to calpain cleavage, normalized to GAPDH. 3–6 optic nerves are included at each time point. Error bars represent the standard error of the mean (SEM). Differences are considered significant with *P < 0.05, **P < 0.01, ***P < 0.001 by one-way ANOVA and Dunnett’s test. (**c**) Overview images (20×) of a native uncrushed optic nerve (control) and an optic nerve at 1 h after crush immunostained against cleaved spectrin. The antibody against cleaved spectrin selectively detects the calpain-specific BDP of spectrin. Below, quantification of the staining intensity along the longitudinal sections of native optic nerves and optic nerves at 1 h after crush. (**d**) Higher magnification micrographs of the representative areas immunostained against cleaved spectrin and the axonal marker smi31 in a native optic nerve and within 300 μm of the crush site in an optic nerve at 1 h after crush. Below, quantification of the intensity of intra-axonal cleaved spectrin up to 300 μm proximal and distal to the crush site. 3 optic nerves per group. *P < 0.05 by independent samples t-test.

The proteolytic activity of the calcium-dependent protease calpain can be assessed by the analysis of the 145 kDa breakdown product (BDP) of spectrin (α-fodrin), which is derived specifically by calpain cleavage^18^. Optic nerve protein lysates were prepared of two regions comprising 1 mm proximal or distal from the crush site at different time points after ONC. An immunoblot for spectrin was performed and expression levels of the 145 kDa spectrin-BDP were quantified as a marker for calpain activation. On both proximal (at 360 min: *P* < 0.001) and distal (at 360 min: *P* < 0.001) sides of the crush, the levels of 145 kDa spectrin-BDP significantly increased after ONC compared to the native optic nerves (Fig. 1a,b). The increase of cleaved spectrin levels started already at 5 min and continued up to 6 h after ONC (Fig. 1a,b). The stable levels of intact spectrin despite the increase of its BDPs are most probably due to its well-known rapid intracellular reproduction with a half-life of 15–40 min^19^. While the total protein levels of calpain were not changed at 360 min after ONC, the levels of the proteolytically active isoform of calpain (76 kDa) were increased in relation to the pro-enzymatic isoform of calpain (80 kDa)^20^ at 6 h after ONC compared to before crush (Supplementary Figure S4).

In order to confirm these results and to specify the localization of calpain activation during AAD, optic nerve sections were prepared for immunohistochemistry using an antibody that specifically recognizes the calpain-induced spectrin-BDP^21^. Already at one hour (h) after ONC, there was a significant increase in staining intensity of the calpain-induced spectrin-BDP in an area 300 μm proximal (*P* = 0.043) and distal (*P* = 0.025) to the lesion site (Fig. 1c). This area corresponds to the region morphologically affected by AAD^10^. The increase in spectrin-BDP could be localized specifically to the axons (Fig. 1d). These results demonstrate that calpain is activated early after ONC in axons during AAD.

### Calpain inhibition attenuates acute axonal degeneration *in vivo*

**Figure 2:**
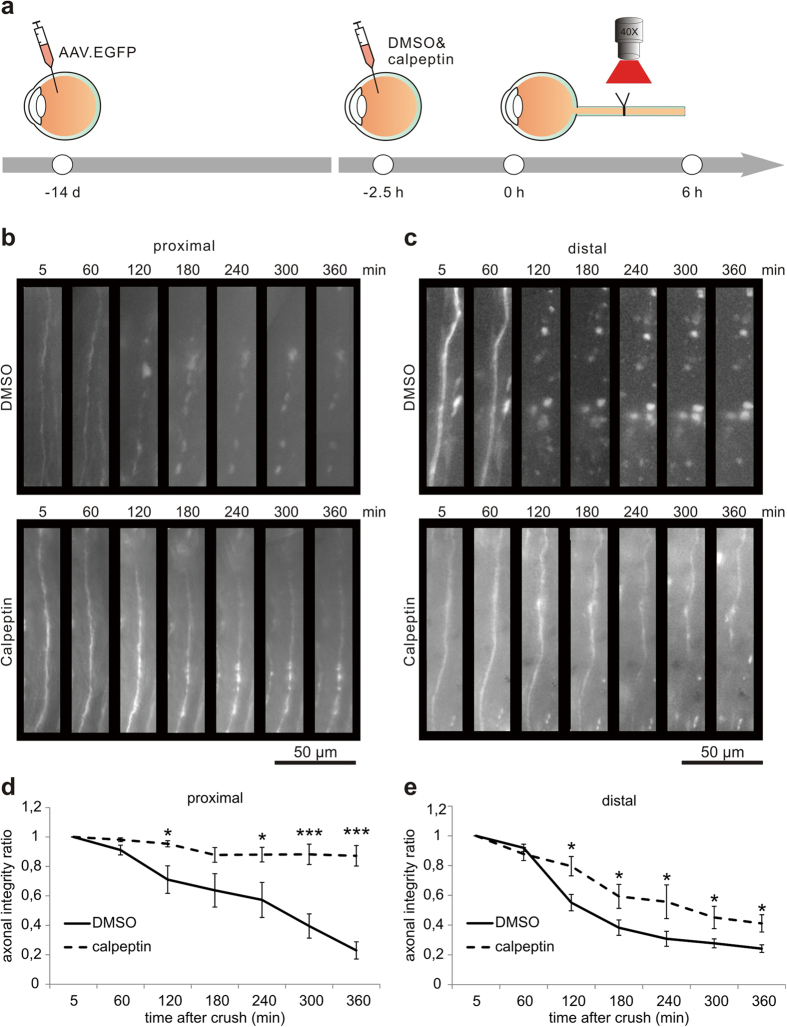
Calpain-inhibition attenuates acute axonal degeneration in the optic nerve *in vivo.* (**a**) AAV.EGFP was injected intravitreally to label the axons of retinal ganglion cells. After two weeks, calpeptin in 7% DMSO or 7% DMSO as control was injected intravitreally 2.5 h before optic nerve crush. Single axons in the optic nerve were imaged within 400 μm from the lesion site before crush and up to 6 h after crush. (**b,c**) Representative images of axonal changes proximal (**b**) and distal (**c**) to the crush site at the indicated time points after crush and previous calpeptin treatment compared to controls. (**d,e**) Quantification of axonal integrity ratios proximal (**d**) and distal (**e**) to the crush site at the indicated time points after crush after calpeptin treatment compared to control. The axonal integrity ratio is the sum of the lengths of the axon fragments at a given time-point divided by the initial length of the axon. 5–6 animals per group. Error bars represent the standard error of the mean (SEM). *P < 0.05, ***P < 0.001 by independent samples t-test.

As calpain was activated early during AAD, we determined whether calpain inhibition would interfere with AAD after ONC. We employed the calpain inhibitor calpeptin^22^ that was injected intravitreally 2.5 h before ONC. Effective calpain inhibition was confirmed by Western blot analysis and revealed a significantly attenuated increase of spectrin-BDP in optic nerve lysates (crush + DMSO & crush + cal proximal: *P* = 0.021; crush + DMSO & crush + cal distal: *P* = 0.011) (Supplementary Figure S1). To assess AAD, we performed an *in-vivo* live imaging of the rat optic nerve following ONC as described before^16^. AAV.hSyn-EGFP was injected intravitreally 2 weeks before imaging to visualize the axons in the optic nerve (Fig. 2a). 2.5 h before ONC, either calpeptin in 7% DMSO or 7% DMSO (control) was injected intravitreally. Live imaging in the anesthetized rat was performed in the area 400 μm proximal and distal to the crush site at different time-points before and up to 6 h after ONC. For each labeled axon, the axonal integrity ratio (AIR) was quantified, which is defined as the sum of the lengths of the remaining axonal fragments divided by the initial length of the axon. A higher AIR thus represents a more intact axon during AAD^10^,^16^. On both proximal (at 360 min: *P* < 0.001) and distal sides (at 360 min: *P* = 0.006) of the crush, calpeptin treatment significantly attenuated the process of AAD after ONC compared to control treatment (Fig. 2). This attenuation by calpeptin was sustained over the whole examination period up to 360 min after ONC. The axon stabilizing effects of calpeptin were, however, more pronounced on the proximal side (AIR at 360 min: control: 0.23 ± 0.06; calpeptin: 0.87 ± 0.07) as compared to the distal side (AIR at 360 min: control: 0.24 ± 0.03; calpeptin: 0.41 ± 0.06) (Fig. 2).

Taken together, these experiments show that calpain inhibition significantly attenuates crush-induced AAD, especially on the proximal side.

### CRMP2 is a downstream target of calpain during acute axonal degeneration

**Figure 3:**
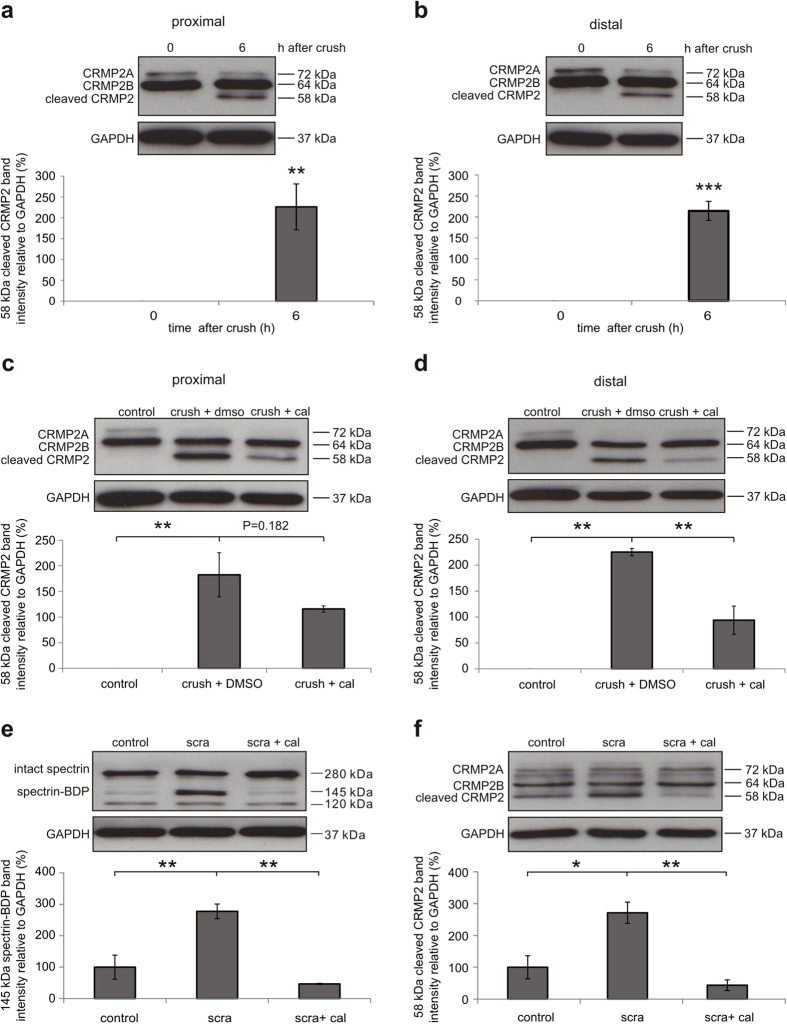
Calpain-mediated cleavage of CRMP2 during acute axonal degeneration *in vivo* and after scratch *in vitro*. (**a,b**) Representative Western blots of CRMP2 proximal (**a**) and distal (**b**) to the crush site in uncrushed native optic nerves (0 h) and in optic nerves at 6 h after crush. Below, quantification of cleaved CRMP2 band intensity relative to GAPDH. 4 optic nerves per group. Error bars represent standard error of the mean (SEM). **P < 0.01, *** *P* < 0.001 by independent samples t-test. (**c,d**) Representative Western blots of CRMP2 proximal (**c**) and distal (**d**) to the crush site in uncrushed native optic nerves (control), in optic nerves at 6 h after crush pretreated with 7% DMSO (crush + DMSO), and in optic nerves at 6 h after crush pretreated with 10 mM calpeptin in 7% DMSO (crush + cal). Cal = Calpeptin. Quantifications of cleaved CRMP2 band intensity relative to GAPDH are shown below. Four optic nerves are included in each group. Error bars represent the standard error of the mean (SEM). **P < 0.01 by one-way ANOVA and Dunnett’s test. (**e,f**) Representative Western blots of spectrin (**e**) and CRMP2 (f) in unscratched primary cortical neuron cultures pretreated with 0.1% DMSO (control), scratched cultures pretreated with 0.1% DMSO (scra + DMSO), and scratched cultures pretreated with calpeptin in 0.1% DMSO (scra + cal). Cal = calpeptin. Sca = scratch. Below, band intensities of 145 kDa cleaved spectrin (**e**) and cleaved CRMP2 (**f**) were quantified and normalized to GAPDH. 3 independent cultures per group. Error bars represent the standard error of the mean (SEM). *P <  < 0.05, **P < 0.01 by one-way ANOVA and Dunnett’s test.

To identify functionally relevant downstream molecular targets of calpain activation during AAD, we evaluated the expression levels of different proteins that had previously been reported to be cleaved by calpain in other model systems^23^,^24^,^25^,^26^,^27^,^28^. Based on previous studies of our group and others^1^,^10^, we focused on proteins that are involved in autophagy, cytoskeleton integrity and axonal transport as these processes are pivotal for axonal degeneration. Protein levels were compared in optic nerve lysates before and 6 h after ONC on the proximal and distal sides of the crush (Supplementary Figure S2). We could not detect any changes in expression levels of the autophagy related proteins beclin-1 or autophagy protein 5 (ATG5). The microtubule-associated protein 2 (MAP-2) was significantly decreased on the proximal side (*P* = 0.034), while tau levels were unaffected. The levels of dynein, which is the major motor protein of retrograde axonal transport^29^, were also not changed during AAD (Supplementary Figure S2). CRMP2 has been described earlier as an important protein linking axonal transport to the cytoskeleton^30^,^31^,^32^,^33^. Interestingly, we found a significant increase of cleaved CRMP2 on both proximal (*P* = 0.006) and distal sides (*P* < 0.001) at 6 h after ONC compared to the native optic nerve (Fig. 3a,b). As CRMP2 cleavage was the most prominent effect observed in our analysis, we next explored the role of CRMP2 for AAD in more detail.

To investigate whether the cleavage of CRMP2 is mediated by calpain activation, we evaluated the effect of the calpain inhibitor calpeptin on the levels of cleaved CRMP2 at 6 h after ONC. Calpeptin treatment indeed attenuated the increase of cleaved CRMP2 *in vivo* at 6 h after ONC in both proximal and distal parts of the optic nerve (proximal: ONC + DMSO: 182.5 ± 42.9%; ONC + cal: 115.8 ± 6.1%; *P* = 0.182; distal: ONC + DMSO: 225.3 ± 7.0%; ONC + cal: 93.9 ± 27.2%; *P* = 0.002) (Fig. 3c,d). This effect was confirmed in lesioned primary cortical neurons *in vitro*. Here, a scratch lesion^34^ was performed 1 h after addition of calpeptin to the culture medium. Cell lysates were collected 6 h after scratch. Compared to control, calpeptin treatment significantly suppressed calpain activation (scra & scra + cal: *P* = 0.001) as well as the cleavage of CRMP2 (scra & scra + cal: *P* = 0.004) at 6 h after scratch as assessed by Western blot of spectrin and CRMP2 (Fig. 3e,f).

### Overexpression of CRMP2 delays bulb formation after axotomy *in vitro*

**Figure 4:**
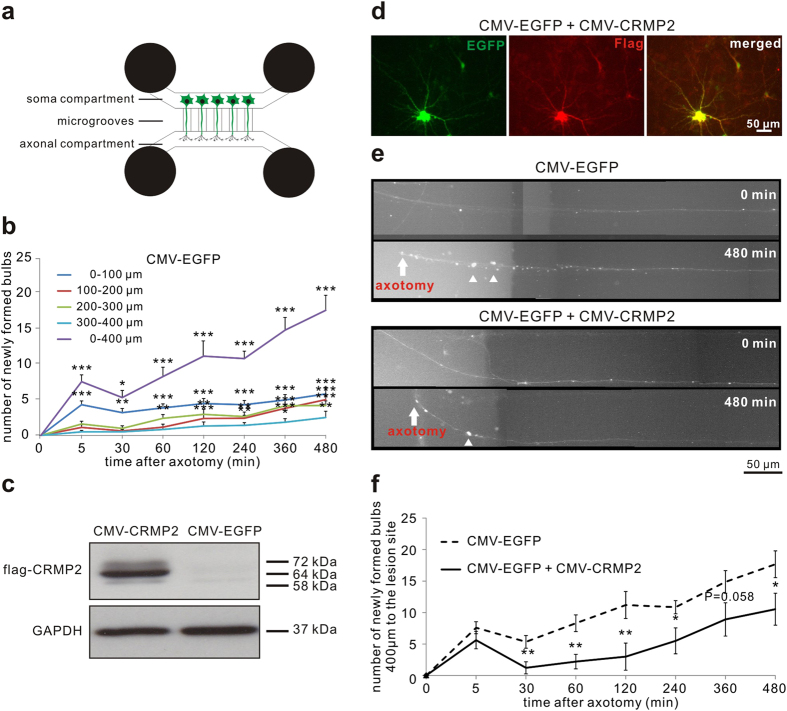
Effects of CRMP2 overexpression on axonal degeneration after axotomy of cortical neurons *in vitro.* (**a**) The microfluidic chamber system consists of 4 wells and two channels. The two channels are connected by microgrooves. Rat cortical neurons are plated in the channel named ‘soma compartment’. Only axons can grow across the microgrooves to enter the other channel called ‘axonal compartment’. (**b**) Quantification of newly formed bulbs in the axons in the given areas proximal to the lesion site at the indicated time points after axotomy after transfection of p.CMV-EGFP. Error bars represent the standard error of the mean (SEM). N ≥ 19 axons. *P < 0.05, **P < 0.01, ***P < 0.001 by one-way ANOVA and Dunnett’s test. (**c**) Representative Western blot with anti-flag antibody in cortical neurons after transfection with p.CMV-EGFP or p.CMV-CRMP2-flag confirming the overexpression of the CRMP2-flag transcript from the plasmid. (**d**) Immunostaining with anti-flag antibody confirming co-transfection of p.CMV-CRMP2-flag and p.CMV-EGFP in the cortical neurons. (**e**) Representative axons proximal to the lesion site at 480 min after axotomy and previous transfection with p.CMV-EGFP as control or co-transfection with p.CMV-CRMP2-flag and p.CMV-EGFP. The number of bulbs was decreased in the axon overexpressing CRMP2 compared to control. (**f**) Quantification of the number of newly formed axonal bulbs within 400 μm proximal to the lesion site at the indicated time points after axotomy and previous transfection with p.CMV-EGFP or both p.CMV-CRMP2-flag and p.CMV-EGFP. N ≥ 19 axons per group. Error bars represent the standard error of the mean (SEM). *P < 0.05, **P < 0.01 by independent samples t-test.

Given the calpain-induced cleavage of CRMP2 during AAD *in vitro* and *in vivo*, we speculated that an increase in uncleaved CRMP2 might have a protective effect on axonal integrity. To examine this hypothesis, we modeled AAD *in vitro*. Primary rat cortical neurons were cultured in a microfluidic chamber that separates axons from somata and dendrites^35^. On DIV8, an axotomy was performed and the axonal changes proximal to the lesion site were imaged live in a conditioned observation chamber over 8 h after axotomy. Interestingly, axotomy in the microfluidic chamber did not result in a complete fragmentation of the axons as observed during AAD *in vivo*, but led to the formation of axonal bulbs in a time-dependent manner. Since bulb formation is also known to be an early sign of axonal degeneration^1^, we quantified the number of newly formed bulbs in single axons within 400 μm proximal to the lesion site after axotomy. We observed a time-dependent significant increase of axonal bulbs as a correlate for axonal degeneration (at 480 min: *P* < 0.001) (Fig. 4b).

For overexpression of CRMP2, we used a plasmid overexpressing human CRMP2 with a flag-tag under control of a CMV promoter (p.CMV-CRMP2-flag)^36^. Overexpression of CRMP2 was confirmed by Western blot against flag (Fig. 4c). To visualize axonal morphology during live-imaging, cortical neurons were co-transfected with both CMV-CRMP2-flag plasmid and a plasmid expressing EGFP (p.CMV-EGFP). Co-transfection was confirmed by immunocytochemistry against anti-flag antibody (Fig. 4d). As control, cortical neurons were transfected with p.CMV-EGFP alone. In both groups, we quantified the number of newly formed bulbs in EGFP-fluorescent axons in an area of 400 μm proximal to the lesion site after axotomy. In the axons overexpressing CRMP2, the number of bulbs was significantly reduced compared to control group at all-time points later than 30 min after axotomy (at 30 min: control: 5.3 ± 1.0; CRMP2: 1.2 ± 1.0; *P* = 0.006; at 240 min: control: 10.8 ± 1.1; CRMP2: 5.5 ± 2.1; *P* = 0.017; at 480 min: control: 17.7 ± 2.1; CRMP2: 10.5 ± 2.5; *P* = 0.036) (Fig. 4e,f). The significantly reduced number of bulbs compared to control was sustained over 480 min after axotomy although both groups continued to develop more bulbs over time (Fig. 4e,f).

### Axonal transport of mitochondria is transiently impaired after axotomy *in vitro*

**Figure 5:**
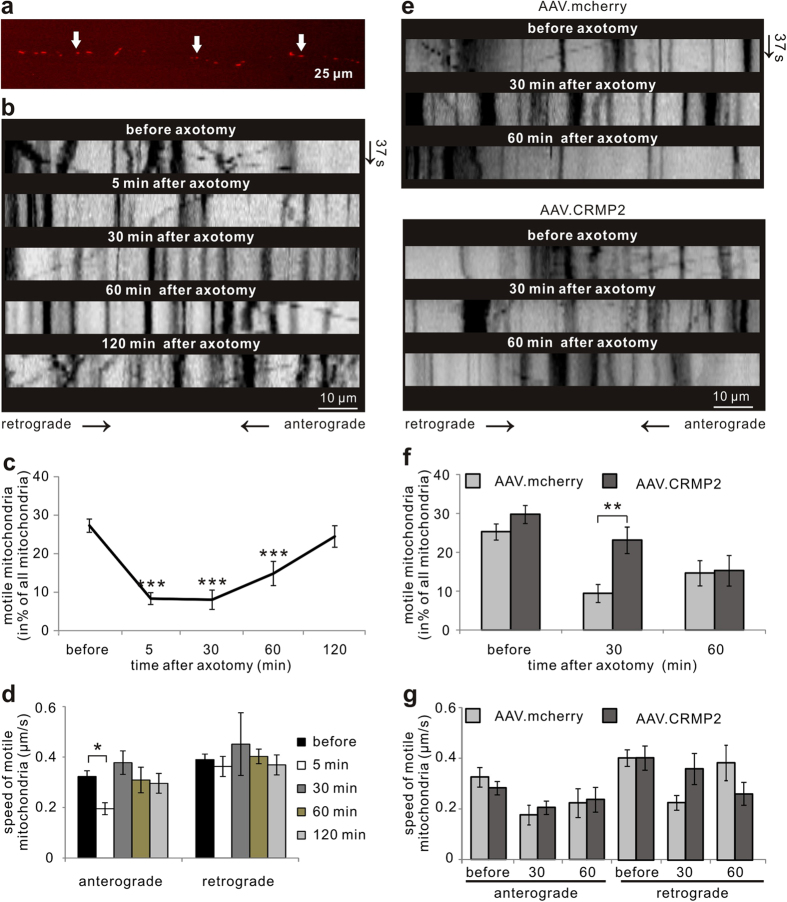
Live imaging of mitochondrial transport after axotomy in cortical neurons *in vitro.* (**a**) Mitochondria labeled with AAV.mito-RFP (arrows) are visualized along the axon. (**b**) Representative kymographs of mitochondrial movements along the axons within 100 μm proximal to the lesion site before and after axotomy during a recording time of 37 s (y-axis). (**c,d**) Quantification of mitochondrial movements in the axon segment 100 μm proximal to the lesion site before and after axotomy. 18–20 axons from >3 independent experiments are included. Error bars represent the standard error of the mean (SEM). ***P < 0.001 by one-way ANOVA and Dunnett’s test. (**e**) Representative kymographs of mitochondrial movements within 100 μm proximal to the lesion site before and after axotomy after transduction with AAV.mcherry or AAV.CRMP2 during a recording time of 37 s (y-axis). (**f,g**) Quantification of mitochondrial movements in the axon 100 μm proximal to the lesion site before and after axotomy after transduction of AAV.mcherry or AAV.CRMP2. N ≥ 14 axons per group. Error bars represent the standard error of the mean (SEM). **P < 0.01 by independent samples t-test.

Axonal bulbs are hallmarks of degeneration and contain an accumulation of unstructured cytoskeletal proteins and different organelles including mitochondria^10^. Since CRMP2 contributes to the regulation of axonal transport and we now identified CRMP2 as an important regulator of AAD, we further examined its influence on mitochondrial transport in AAD. To study the kinetics of mitochondrial transport after axotomy, we used the microfluidic chamber system. Cortical neurons were transduced with AAV.mito-RFP to visualize mitochondria (Fig. 5a). On DIV8, live imaging of mitochondrial transport was performed after axotomy in single axons 100 μm proximal to the lesion site and quantified by kymographs. The percentage of motile mitochondria decreased significantly within 60 min after axotomy (5 min: 8.3 ± 1.5%; *P* < 0.001; 30 min: 8.0 ± 2.5%; *P* < 0.001; 60 min: 14.8 ± 3.1%; *P* < 0.001) compared to before axotomy (27.3 ± 1.7%) (Fig. 5b,c). However, at 2 h after axotomy (24.5 ± 2.8%), the percentage of motile mitochondria had recovered to the levels before axotomy (Fig. 5b,c). The speed of anterograde mitochondria transport was decreased significantly at 5 min (*P* = 0.035), returning to normal levels already at 30 min after axotomy (before axotomy: 0.32 ± 0.02 μm/s; 5 min: 0.20 ± 0.02 μm/s; 30 min: 0.37 ± 0.05 μm/s) (Fig. 5b,d). The speed of retrograde mitochondrial transport was not significantly altered at the analyzed time-points (Fig. 5b,d).

### Overexpression of CRMP2 temporally rescues mitochondrial transport after axotomy *in vitro*

CRMP2 plays an important role in axonal transport by adapting the motor protein kinesin-1 to transport packets^31^,^33^. Based on our findings that CRMP2 is cleaved by calpain early in AAD, we tested whether overexpression of CRMP2 would rescue the impairment of mitochondrial transport after axotomy. Primary cortical neurons were transduced with AAV overexpressing human CRMP2 and the fluorophore mcherry (AAV.hSyn-CRMP2-mcherry) or only mcherry (AAV.hSyn-mcherry) (control). Overexpression of CRMP2 was confirmed by qRT-PCR analysis (Supplementary Figure S3) and mitochondria were labeled with mitotracker Green. In the unlesioned axons, there were no significant differences in the percentage (AAV.hSyn-mcherry: 25.3 ± 2.0%; AAV.hSyn-CRMP2: 29.8 ± 2.2%) or the speed of motile mitochondria between AAV.hSyn-mcherry and AAV.hSyn-CRMP2-treated cultures (anterograde speed: AAV.hSyn-mcherry: 0.33 ± 0.04 μm/s; AAV.hSyn-CRMP2: 0.28 ± 0.02 μm/s; retrograde speed: AAV.hSyn-mcherry: 0.41 ± 0.03 μm/s; AAV.hSyn-CRMP2: 0.41 ± 0.05 μm/s) (Fig. 5e–g). At 30 min after axotomy, the percentage of motile mitochondrial decreased significantly in the control group (9.4 ± 2.3% of all mitochondria), but overexpression of CRMP2 by AAV.hSyn-CRMP2 almost completely rescued this effect (23.2 ± 3.3% of all mitochondria; *P* = 0.002) (Fig. 5e,f). At 60 min after axotomy, the percentage of motile mitochondria equaled out in both groups (Fig. 5e,f). Interestingly, the speed of motile mitochondria did not show significant differences between both groups at the analyzed time-points (Fig. 5e,g).

### Overexpression of CRMP2 attenuates acute axonal degeneration after optic nerve crush *in vivo*

**Figure 6:**
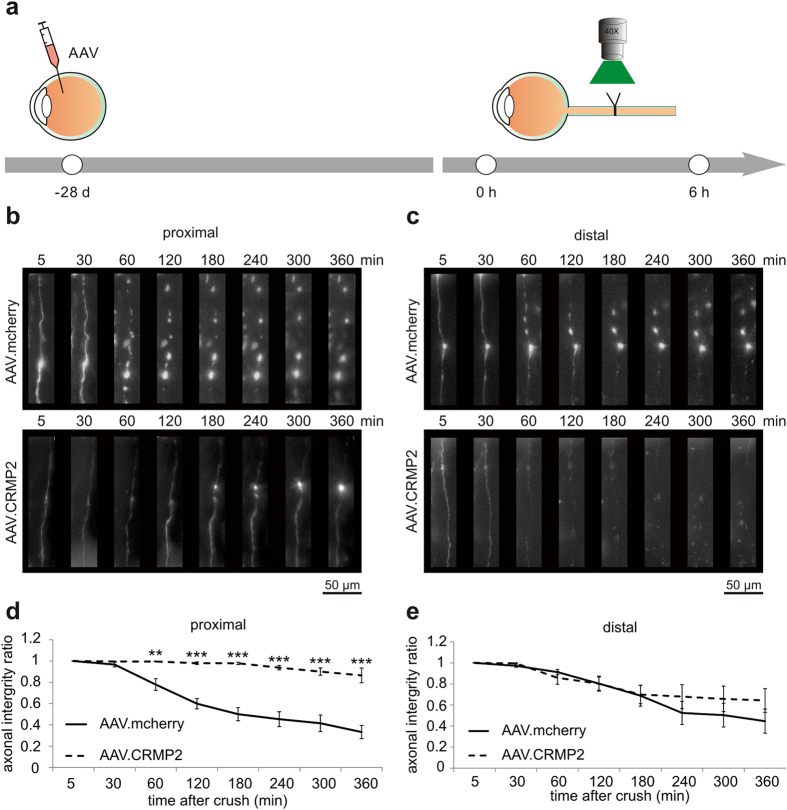
Effects of CRMP2 overexpression on crush-induced AAD *in vivo.* (**a**) Intravitreal injections of AAV.mcherry or AAV.CRMP2 visualized the axons of retinal ganglion cells. After four weeks, single axons within 400 μm of the crush site were imaged before crush and during 6 h after crush. (**b,c**) Representative images of lesioned axons proximal (**b**) and distal (**c**) to the crush site at the given time points after crush and previous transduction with either AAV.CRMP2 or AAV.mcherry (control). In the control group (upper row), a rapid formation of degeneration bulbs and subsequent axonal fragmentation can be observed. These morphological hallmarks of AAD are significantly less pronounced in the axons overexpressing CRMP2 (lower row). (**d**,**e**) Quantification of the axonal integrity ratio proximal (**d**) and distal (**e**) to the crush site at the indicated time points after crush. 5–7 rats per group. Error bars represent the standard error of the mean (SEM). **P < 0.01, ***P < 0.001 by independent samples t-test.

Our findings in the microfluidic chambers system demonstrate that increased levels of CRMP2 counteract bulb formation and impairment of axonal transport after axotomy. To analyze the effects of CRMP2 overexpression on AAD *in vivo*, we transduced RGCs with AAV, resulting in overexpression of CRMP2 in the optic nerve axons and visualized AAD in the living animal. AAV.hSyn-CRMP2 or AAV.hSyn-mcherry was injected intravitreally at 4 weeks before imaging to allow for sufficient transcript expression (Fig. 6a). Overexpression of CRMP2 in transduced rat optic nerves and retinas was confirmed by qRT-PCR and Western blot analysis (Supplementary Figure S3). On the proximal side of the crush, axonal fragmentation during the first 6 h after ONC was almost completely blocked in the axons overexpressing CRMP2 (at 360 min: *P* < 0.001) (Fig. 6b,d). On the distal side of the crush, however, there was no significant difference between the groups and only little protective effects of CRMP2 overexpression on axonal integrity (Fig. 6c,e).

### Proteomics analysis of acute axonal degeneration in the optic nerve

**Figure 7:**
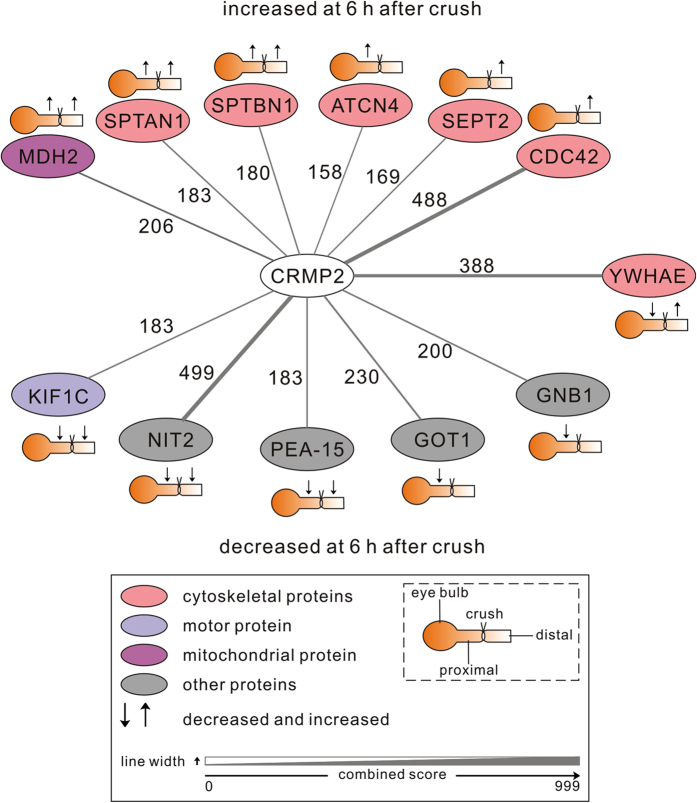
Proteomics analysis of acute axonal degeneration – the regulated CRMP2-interactome. Proteomic analysis of optic nerve lysates from the regions 1 mm proximal and distal to the lesion site revealed that the expression levels of 135 out of 2685 identified proteins were significantly altered at 6 h after crush as compared to control. For these regulated proteins, a STRING (v10 for rat) database search was performed to identify proteins with previous evidence for interaction with CRMP2. The search yielded 12 proteins that are shown in the scheme. Functional protein classes are indicated by the different colors. The significant changes in expression levels (↑increase; ↓decrease) are represented with regards to the specific location proximal or distal to the crush site. Rat gene symbols are displayed for the corresponding proteins. The level of evidence for an interaction of the given protein with CRMP2 is given as the combined score from the STRING database indicated by the edge width of the connecting lines and the adjacent numbers. Abbreviations for the evaluated rat proteins: ACTN4 = alpha-actinin 4; CDC42 = cell division control protein 42; CRMP2 = collapsin response mediator protein-2; GNB1 = G protein beta subunit 1; GOT1 = aspartate transaminase; KIF = kinesin-like protein; MDH2 = malate dehydrogenase 2; NIT2 = omega-amidase; PEA-15 = astrocytic phosphoprotein; SEPT2 = septin 2; SPTAN1 = alpha-II spectrin; SPTBN1 = beta-II spectrin; YWHAE = 14-3-3 protein epsilon.

To identify relevant downstream interaction partners of CRMP2 and further molecular targets that contribute to AAD, we performed a proteomics analysis of optic nerve lysates at 6 h after crush lesion in comparison to the unlesioned optic nerve. Protein lysates were prepared of two regions comprising 1 mm proximal or distal from the crush site or corresponding regions of unlesioned optic nerves. 2685 protein groups were identified in total. Significant differences in expression levels at 6 h after ONC compared to control were detected for 135 proteins. We performed a STRING (v10 for rat) database search^37^ for these regulated proteins and identified 12 proteins with previous evidence for interaction with CRMP2 (Fig. 7 and Supplementary Table S1). Several cytoskeletal proteins that are known to interact with CRMP2 were increased in expression at 6 h after crush. These proteins include the spectrin subunits alpha-II and beta-II on both sides of the crush, the actin-binding protein alpha-actinin 4 (ACTN4) on the proximal side and the cytoskeleton regulators septin 2 (SEPT2) and cell division control protein 42 (CDC42) on the distal side of the crush. Moreover, the mitochondrial enzyme malate dehydrogenase 2 (MDH2), also reported to interact with CRMP2, was more abundant on both sides of the crush. On the other hand, expression levels of several known CRMP2 interactors involved in axonal transport, glutamate metabolism and survival pathways were significantly decreased at 6 h after crush. On both sides of the crush the motor protein kinesin-like protein (KIF) 1 C, the activator of the ERK/MAPK pathway astrocytic phosphoprotein (PEA-15) and the metabolic enzyme omega-amidase (NIT2) were decreased. The proteins aspartate transaminase (GOT1) and G-protein beta subunit 1 (GNB1) were decreased on the proximal side only. The 14-3-3 protein epsilon (YWHAE) was decreased on the proximal side and increased on the distal side of the crush.

## Discussion

In this study, we characterize new molecular mechanisms underlying AAD in the optic nerve *in vivo*. We demonstrate that calpain is activated early after the initial transient calcium influx and that the following cleavage of CRMP2 is a major step in AAD. Both inhibition of calpain and overexpression of intact CRMP2 are protective against lesion-induced AAD. Moreover, our *in vitro* data suggests that CRMP2 exerts its protective function by maintaining axonal mitochondria transport.

Activation of the calcium-sensitive protease calpain was shown before to play a major pathophysiological role in many neurodegenerative diseases^38^. Moreover, several studies in different *in vitro* models and in rat traumatic brain injury have demonstrated that calpain activity is increased early in traumatic axonal degeneration, yet the kinetics appear to depend on the lesion model^39^,^40^,^41^,^42^. In AAD of the rat spinal cord *in vivo*, an activation of calpain was found at 30 minutes after the lesion while calpain inhibitors completely protected the axons from fragmentation up to one hour after lesion^9^. Moreover, the calpain inhibitor calpeptin was shown to decrease neurodegeneration and inflammation in a rat optic neuritis model^43^. In our model, calpain is activated early (within the first 5 minutes after lesion) during AAD of the optic nerve and the activity increases significantly faster on the proximal side than on the distal side of the crush. These differences in calpain activity kinetics precede the subsequently distinct morphological fates of the proximal and distal axon stumps and might contribute to the already established role of calpain and its endogenous inhibitor calpastatin in Wallerian degeneration^44^,^45^.

We found that calpain activation after ONC was localized specifically to the axonal compartment. Interestingly, the increased cleaved-spectrin signal was limited to the area 500 μm proximal and distal to the lesion site, which corresponds to the area prone for fragmentation during AAD. As we could recently show, following an acute axonal lesion in the spinal cord, an increased expression of the autophagy-related proteins ATG5 and ULK1 was observed in the very same area in the vicinity of the lesion, whereas ATG7 was propagated along the axon^46^. The local confinement of molecules involved in axonal degeneration thus establishes the physiological boundaries of AAD and is crucial for the initial integrity of axon parts, which are more remote to the crush site.

Treatment with the calpain inhibitor calpeptin completely stabilized the axons on the proximal side over 6 hours after crush lesion *in vivo* while the effect was less pronounced on the distal side. Calpeptin treatment resulted in only a small but significant decrease of 145 kDa spectrin BDP levels 6 hours after crush whereas CRMP2 cleavage was attenuated more pronounced. Thus the incomplete inhibition of calpain seems to affect the cleavage of different target proteins to different degrees. Nevertheless this seems to be sufficient to attenuate axonal degeneration, e.g. through the stronger inhibition of CRMP2 cleavage and putatively other targets. Calpain inhibition by different drugs, vectors or genetical modifications was shown before to have protective effects in several models of neurodegeneration^45^,^47^,^48^,^49^. However, much like in our current model, calpain-inhibitors were mostly applied before the lesion. Our data on spectrin cleavage shows that calpain-mediated degradation is detectable as early as 5 minutes after the lesion and could therefore not be prevented by calpain inhibition at later time-points. From a translational point of view, calpain-inhibition thus appears not to be a very promising pharmacological target for acute axonal lesions, because in a clinical setting a therapeutic intervention will not be available within the first minutes following the trauma.

To better understand further downstream molecular mechanisms in AAD and to identify other drugable targets, we screened several calpain substrates and detected a marked CRMP2 cleavage by calpain during AAD in the optic nerve. CRMP2 plays an important role in neurite outgrowth and serves as a linker between the cytoskeletal proteins tubulin and actin and the motor protein kinesin, a molecular motor executing anterograde axonal transport^30^,^31^,^50^. Altered CRMP2 has been associated with several neurodegenerative disorders^51^. It has been shown that calpain cleaves CRMP2 in rat cortical neurons and that CRMP2 cleavage products coincide with the appearance of spectrin BDP in rat traumatic brain injury^25^. Our study provides the first evidence that CRMP2 plays a pivotal role in AAD after traumatic optic nerve lesion.

In our *in vitro* experiments in cortical neurons, CRMP2 overexpression significantly delayed the formation of axonal bulbs after axotomy. Cleaved CRMP2 was found to localize preferentially in axonal swellings of lesioned mouse superior cervical ganglia cells^52^. Thus, calpain-mediated cleavage of CRMP2 likely represents a crucial initial molecular step for the formation of degenerative axonal bulbs. As the formation of axonal bulbs is a hallmark of axonal degeneration and is often followed by axonal fragmentation^1^,^2^, our *in vitro* data suggested that CRMP2 overexpression would also counteract axonal degeneration *in vivo*. This was indeed confirmed after crush lesion in the optic nerve, where CRMP2 overexpression protected the proximal part of the axons from degeneration. The magnitude of this effect on the proximal side of the lesion was similar to the one elicited by application of calpeptin. CRMP2 thus appears to be a pivotal downstream target of calpain during AAD within the first 6 hours after lesion. Because we observed a strong attenuation of axonal degeneration through overexpression of intact CRMP2, the degenerative cascade appears to be triggered by the degradation and subsequent lack of functional CRMP2. Overexpression of intact CRMP2 did not interfere with the levels of cleaved CRMP2 and it is therefore less likely that axonal degeneration is influenced by cleavage products of CRMP2. Notably, we could not detect any significant cleavage of other predicted calpain target proteins within the first 6 h of AAD in the optic nerve, except for a degradation of MAP2, which was, however, significant only on the proximal side. The cleavage of MAP2 is probably a first sign of the calpain-mediated cleavage of cytoskeletal proteins that has been observed at 5 days after optic nerve transection^44^.

Interestingly, AAD was inhibited much more pronounced on the proximal side of the crush by both calpain inhibition and CRMP2 overexpression. Similar findings were obtained earlier with ROCK2-downregulation^53^. In our opinion the difference is most probably caused by the molecular differences that also drive Wallerian degeneration (WD) of the distal axon only. It is now known that WD is initiated in a calcium-indpendent manner by the disturbed supply of the distal axon with survival signals from the soma like NAD^+^ and nicotinamide mononucleotide adenylyltransferase (NMNAT)^12^. We propose that this mechanism adds to the calcium-induced changes of AAD and is not substantially counteracted on the distal side by calpain-inhibition or CRMP2-overexpression.

Impairment of axonal transport and mitochondrial dysfunction has been previously implicated in axonal degeneration^54^. Here, we imaged the kinetics of mitochondrial transport after axotomy *in vitro*. We found that the percentage of motile mitochondria was decreased only within the first hour after axotomy. Moreover, the speed of anterograde transport was decreased at 5 minutes after axotomy. Interestingly, both the percentage of motile mitochondria and the transport speed recovered to normal levels as soon as one hour after axotomy. Thus, the initial increase in calpain activity did not result in a persistent damage to axonal transport mechanisms. Mechanistically, persistent axonal transport may even be required for the formation of axonal bulbs, allowing to transport axonal cargo to these local swellings.

CRMP2 plays an important role in axonal transport through its direct molecular interaction with the motor proteins kinesin and dynein^31^,^33^. Calpain-mediated cleavage of CRMP2 impairs its binding to kinesin^25^. We show here that CRMP2 overexpression not only inhibits bulb formation but at the same time rescues the impairment of axonal mitochondrial transport that is observed within the first hour after axotomy. It is thus very likely that the cleavage of CRMP2 by calpain and the resulting impairment of its binding to motor proteins are responsible for the breakdown of axonal transport. Impaired axonal transport then results in a local accumulation of organelles and finally the formation of axonal bulbs, as we have observed previously^10^.

Proteomic studies of axonal degeneration in the optic nerve have been performed previously at later time-points, 24 and 48 h after ONC^55^. Here, we describe differences in protein expression during early AAD at 6 h after ONC compared to control. We focused on regulated proteins that have been previously linked to CRMP2. Most notably, the motor protein KIF1C was reduced on both sides of the crush. KIF1 is essential for axonal transport and it was shown before that CRMP2 binds KIF1 to form a complex that is involved in tubulin trafficking to the distal axon^31^. Therefore, the decreased levels of KIF1 will augment the negative impact on axonal transport caused by CRMP2 cleavage. Interestingly, expression levels of several cytoskeletal proteins including spectrin and actinin were increased on both sides of the crush. As cytoskeletal proteins are continuously transported along the axon, the block of axonal transport around the lesion might lead to this accumulation. Such a rapid accumulation around the crush has been demonstrated before for synaptic proteins in the optic nerve^56^. Similarly, the increase in the mitochondrial protein MDH2 might reflect the accumulation of mitochondria in the area of AAD due to impaired axonal transport. The decreased levels of PEA-15, which plays a role in the ERK/MAPK signaling pathway regulating neuronal cell survival^57^, and of GOT1, NIT2 and GNB1, which are involved in glutamate metabolism and signal transduction^58^, might contribute to the degeneration of the axons, but still need to be further evaluated.

In conclusion, this study demonstrates that calpain controls AAD by cleaving CRMP2 and consequently affecting mitochondrial transport. Both calpain inhibition and CRMP2 overexpression markedly attenuate the process of AAD on the proximal side of the lesion. Besides CRMP2, an early regulation of cytoskeletal proteins and proteins involved in axonal transport and glutamate metabolism is identified by proteomic analysis. We propose that these proteins represent novel therapeutic targets in traumatic and degenerative diseases of the CNS.

## Methods

### Plasmids and adeno-associated viral vectors production

The following plasmids and AAV were used: p.CMV-CRMP2-flag (generous gift from Mahnaz Moradi-Améli, Université Lyon 1, France)^36^, p.CMV-EGFP (Genbank ID: KT343252), pAAV.hSyn-CRMP2 [Genbank ID: KT345944], pAAV.hSyn-mcherry [Genbank ID: KT345943], AAV.hSyn-EGFP^59^ and AAV.hSyn-mito-RFP [Genebank ID: KT358727]. The plasmids pAAV.hSyn-CRMP2 and pAAV.hSyn-mcherry contain two hSyn-promoters. Detailed cloning procedures are provided in the supplementary methods.

For all experiments, adeno-associated virus of the pseudotype 1/2 was used. AAV of the hybrid serotype 2/1 were generated consisting of AAV2 inverted terminal repeats (ITR) packed into AAV1/AAV2 hybrid capsids (molar ratio 1:1). Both AAV-hSyn-CRMP2 and AAV.hSyn-mcherry were produced using the pACG-2 helper-plasmid (kindly provided by Arun Srivastava, University of Florida, USA) resulting in the hybrid serotype AAV1/mutAAV2^60^. Details of AAV-production are described in the supplementary methods.

### Animal experiments

All animals were treated according to the guidelines and regulations of the local animal research council and legislation of the State of Lower Saxony, Germany. All animal experiments were approved by the Lower Saxony State Office for Consumer Protection and Food Safety (LAVES).

### Neuronal cell culture, nucleofection, viral transduction and scratch assay

Primary rat cortical neurons were prepared from embryonic day 18 (E18) rats as described elsewhere^61^. Briefly, dissected cortices were trypsinized for 10 min at 37 °C, and then triturated. After centrifugation, cells were suspended in Neurobasal medium supplemented with B-27, L-Glutamine, transferrin and PSN.

Neurons were transfected by nucleofection (Lonza; program O-005) with 4 μg p.CMV-EGFP or co-transfected with 2.5 μg p.CMV-CRMP2-flag and 1.5 μg p.CMV-EGFP and seeded in 6-well plates or microfluidic chambers.

For viral transduction, 1.5 × 10^5^ cells were transduced with 5 × 10^6^ TU AAV.hSyn- CRMP2-mcherry, 1 × 10^6^ TU AAV.hSyn-mcherry or 1 × 10^7^ TU AAV.mito-RFP at 4 h after seeding, resulting in equal transduction rates (90% of all cells) and only minor toxicity.

The scratch assay was performed with a 200 μl pipette tip in a 48-well plate (1.5 × 10^5^ cells per well) on DIV8.

### Microfluidic chambers, axotomy and live imaging of axonal degeneration *in vitro*

Microfluidic chambers were produced based on previously published protocols^35^,^62^ as described in the supplementary method. Cortical neurons were seeded into one main channel named ‘soma compartment’. In each chamber, 3 × 10^5^ cells were seeded after nucleofection or 1.5 × 10^5^ cells for viral transduction. After 7 days *in vitro*, axons grew across the microgrooves and entered the other main channel called ‘axonal compartment’.

Axotomy was performed by a gentle vacuum suction in the axonal compartment. Axons were cut when the air bubble passed through the compartment as confirmed by microscopy.

For live imaging of axonal degeneration in the chambers, EGFP fluorescent axons were imaged in a microscope incubation system (37 °C, 5% CO_2_) attached to an inverted microscope (Axiovert, Zeiss) at 40x magnification. The axons were imaged before axotomy and at different time points after axotomy. Only lesioned axons were included for evaluation.

### Live imaging of axonal mitochondria transport *in vitro*

Live imaging of axonal mitochondria transport was performed in microfluidic chambers. To check the kinetics of mitochondrial transport after axotomy, AAV.mito-RFP was used to visualize mitochondrial transport. To check the effect of CRMP2 on mitochondrial transport, the cells were first transduced with AAV.hSyn-CRMP2-mcherry or AAV.hSyn-mcherry. 100 nmol MitoTracker Green FM (Invitrogen) were then added in each chamber to label mitochondria before imaging. A time-lapse movie of mitochondria was taken for 37 s with 500 ms exposure time at 40x magnification. Mitochondrial movement in the axons was quantified within 100 μm proximal to the lesion site. Moving mitochondria were defined as having a speed of at least 0.07 μm/s. The movies were quantified with the ImageJ plugin MultipleKymograph. At least 6 chambers were included for each group.

### Western blot analysis

The following primary antibodies were employed: anti-spectrin (1:500; BML-FG6090; Enzo), anti-CRMP2 (1:1000; #9393; Cell signaling), anti-ATG5 (1:300; AP1812b; Abgent), anti-beclin-1 (1:1000; #3738; Cell signaling), anti-MAP-2 (1:1000; MAB3418; Millipore), anti-tau (1:1000; T9450; Sigma), anti-dynein IC (1:500; MMS-400P; Covance), anti-flag M2 antibody (1:1000; F1804; Sigma), and GAPDH (1:2500; G9545; Biotrend). The following HRP-coupled secondary antibodies were used: anti-mouse HRP (7076P2; Cell signaling), anti-rabbit HRP (7074P2; Cell signaling). Detailed Western blot procedures are provided in the supplementary method.

### Immunohisto- and -cytochemistry

For immunohistochemistry, dissected optic nerves were fixed in 4% PFA at 4 °C overnight and then incubated with 30% sucrose at 4 °C for at least 48 h. Longitudinal sections (16 μm thick) were dried at 37 °C for 20 min, and rehydrated in 0.05 M Tris/1.5% NaCl (pH 7.6) at RT for 45 min. Antigen retrieval was performed in 0.05 M Tris/1.5% NaCl (pH 9) at 60 °C for 4 h. Afterwards, sections were permeabilized in methanol at −20 °C for 10 min. After incubation in a blocking reagent (Dako Diluent) at RT for 30 min, primary antibodies were applied at 4 °C overnight. Primary antibodies against Smi31 (1:1000; SMI-31R; Covance), cleaved spectrin (1:5000; a generous gift from Dr Robert Siman, University of Pennsylvania) was used. After washing, sections were incubated with secondary antibody at RT for 1 h. After incubation in DAPI at RT for 10 min, the sections were mounted with Mowiol (Sigma-Aldrich). Micrographs were taken with an AxioPlan microscope (Zeiss) at 20x magnification. The intensity of cleaved spectrin along the optic nerve was obtained using the line plot profile tool of ImageJ 1.49a. For quantification of the intensity of cleaved spectrin in the axons, 8 pictures were taken for proximal and distal parts separately at 63x magnification using the pseudo-confocal ApoTome device (Zeiss). These pictures covered the area of 300 μm proximal and distal to the crush site in the longitudinal optic nerve sections. In total, 5 sections from 3 optic nerves in each group were evaluated.

For immunocytochemistry, cortical neurons were fixed in 4% PFA at RT for 10 min, and then permeabilized in 0.25% triton-100 at RT for 10 min. After blocking with 5% goat serum at RT for 30 min, cells were incubated with primary antibody against flag M2 (1:500; F1804; Sigma) at 4 °C overnight. Afterwards, the goat anti-mouse cy3 secondary antibody (Dianova) was applied at RT for 1 h. Finally, cells were counterstained with DAPI at RT for 10 min and mounted with Mowiol.

### Intravitreal injections, optic nerve crush and *in vivo* live imaging

Intravitreal injections were performed with a Hamilton syringe. All substances were injected in a total volume of 5 μl per eye. 10 mM Calpeptin (Calbiochem) in 7% DMSO or 7% DMSO in deionized H_2_O as control were injected 2.5 h before ONC. AAV were injected 2–4 weeks before live-imaging in previously optimized titers resulting in equal transduction rates of the retina with no obvious toxicity (AAV.hSyn-EGFP: 1.7 × 10^8^ transforming units (TU); AAV.hSyn-mcherry: 2.8 × 10^8^ TU; AAV.hSyn-CRMP2: 2.1 × 10^8^ TU per eye).

Optic nerve surgery and live imaging were carried out as reported before^16^. Briefly, a median skin incision was made between both eyes. The lacrimal gland was moved to the front and connective tissue removed. The eye was rotated by pulling the superior rectus muscle carefully. The optic nerve was exposed by a longitudinal incision of the surrounding dura. For crush lesion, a surgical suture (Ethicon, 10–0 Ethilon) was constricted tightly around the optic nerve for 30 s. For live imaging, the rat was transferred to a Zeiss Examiner microscope adapted with a 40x water immersion objective. Animals were kept under anesthesia based on constant monitoring of oxygen saturation, heart rate and signs of awakening. Z-stack images were taken of the area 400 μm proximal and distal to the crush site before and after ONC. Images were processed using AxioVision 4.8 (Zeiss) and CorelDraw X3 software. For each axon, the axonal integrity ratio was quantified, which is calculated by dividing the sum lengths of all axon fragments at a given time-point by the initial length of the axon.

### Proteomics analysis

Optic nerves were dissected in the regions 1 mm proximal and distal to the crush site at 6 h after ONC. The samples were frozen, homogenized, sonificated and centrifuged as described for Western blot in the supplementary method. To minimize variations due to experimental conditions or intra-animal variability, 4 independent optic nerves per group were pooled into one sample. Protein concentration was measured by BCA assay. Samples were reconstituted in 1xNuPAGE LDS Sample Buffer (Invitrogen) and separated on 4–12% NuPAGE Novex Bis-Tris Minigels (Invitrogen). Gels were stained with Coomassie Blue for visualization purposes, and each lane sliced into 23 equidistant parts regardless of staining. After washing, gel slices were reduced with dithiothreitol (DTT), alkylated with 2-iodoacetamide and digested with trypsin overnight. The resulting peptide mixtures were extracted, dried in a SpeedVac, reconstituted in 2% acetonitrile/0.1% formic acid/(v:v) and prepared for nanoLC-MS/MS as described previously^63^. Further detailed information about mass spectrometric analysis and data processing is provided in the supplementary method.

### Statistical analysis

Statistical comparisons between two groups were performed using independent samples t-test, whereas multiple group comparisons were performed using one-way ANOVA followed by Dunnett’s post-hoc test. Significances are indicated with *P < 0.05, **P < 0.01, ***P < 0.001. All data are shown as mean ± standard error of the mean (SEM). All experiments were performed at least in triplicate.

## Additional Information

**How to cite this article**: Zhang, J.-N. *et al.* Calpain-mediated cleavage of collapsin response mediator protein-2 drives acute axonal degeneration. *Sci. Rep.* **6**, 37050; doi: 10.1038/srep37050 (2016).

**Publisher’s note:** Springer Nature remains neutral with regard to jurisdictional claims in published maps and institutional affiliations.

## Supplementary Material

Supplementary Information
