# Supplementary material for: Calpain-mediated cleavage of collapsin response mediator protein-2 drives acute axonal degeneration
Source: Sci Rep. 2016 Nov 15;6:37050. doi: 10.1038/srep37050 (PMC5109185; doi:10.1038/srep37050)
Supplement: Supplementary Information [file srep37050-s1.doc]

**Supplementary Information**

**for**

**Calpain-mediated cleavage of collapsin response mediator protein-2 drives acute axonal degeneration**

**Authors:** Jian-Nan Zhang^1^, Uwe Michel^1^, Christof Lenz^2,3^, Caroline C. Friedel^4^, Sarah Köster^5,6^, Zara d’Hedouville^1^, Lars Tönges^1^, Henning Urlaub^2,3^, Mathias Bähr^1,6^, Paul Lingor^1,6Ŧ^, Jan C. Koch^1,6Ŧ*^

^1^Department of Neurology, University Medicine Göttingen, 37075 Göttingen, Germany

^2^Bioanalytical Mass Spectrometry, Max Planck Institute for Biophysical Chemistry, 37077 Göttingen, Germany.

^3^Institute of Clinical Chemistry, University Medicine Göttingen, 37075 Göttingen, Germany.

^4^Institute for Informatics, Ludwig-Maximilians-University Munich, 8033 Munich, Germany.

^5^Institute for X-Ray Physics, Georg-August-University Göttingen, 37077 Göttingen, Germany.

^6^Center for Nanoscale Microscopy and Molecular Physiology of the Brain (CNMPB), 37075 Göttingen, Germany.

^Ŧ^ These authors contributed equally to this work.

^*^Correspondence and requests for materials should be addressed to J.C.K. (email: jkoch@med.uni-goettingen.de)

**Supplementary Methods:**

**Cloning of plasmids**

For cloning of **pAAV.hSyn-mito-RFP**, the insert from pTurboRFP-mito (Evrogen) containing a mitochondrial targeting sequence (MTS) and the red fluoresencent protein (RFP) was ligated into pBS-Shuttle at the NheI and NotI restriction sites. The insert containing MTS and RFP was then cut from the shuttle vector with a NheI and SalI digestion and subcloned into pEGFP-N2 (Clontech). Next, the MTS and RFP containing insert was transferred from pEGFP-N2 with NheI and Acc65I digestion to pAAV-9(5)hSyn-EGFP-CytBAS-ohneNot ^1^. The backbone was religated after a digestion with BstAPI. The resulting plasmid pAAV-hSyn-TurboRFP-mito was sequenced and used for AAV production.

For cloning of **pAAV.hSyn-CRMP2**, pCMV-CRMP2-flag (gift from Mahnaz Moradi-Améli, Université Lyon 1, France) ^2^ was cut with HindIII and EcoRV, and the CRMP2 fragment was cloned into a HindIII- and HincII- cut pBluescript. The resulting pBSKII-CRMP2 was then cut with XmaI and SacI, and the resulting insert was cloned into an AgeI- and SacI- cut pAAV vector in 3´of an hSyn promoter and 5´of a bGH polyA tail. The resulting vector, pAAV-CRMP2-hsyn-mcherry was sequenced and used for AAV production.

**Adeno-associated viral vectors**

AAV production was performed as described before ^1^. Briefly, HEK293 cells were transfected with calcium phosphate and a plasmid mix consisting of the respective pAAV-expression vector, pHELPER, pAAV-RC and pH21 in a molar ratio of 1:1:0.5:0.5. The plasmids pAAV-RC and pHELPER were from Stratagene, and the plasmid pH21 (pAAV1) expressing AAV serotype 1 capsids, was kindly provided by Helen Fitzsimons (Neurologix, Inc. OSU Comprehensive Cancer Center, Columbus, USA) and Matthew During (Molecular Virology, Immunology, and Medical Genetics, Columbus, USA). For production of AAV-hSyn-CRMP2 and AAV.hSyn-mcherry, the pACG-2 helper-plasmid (kindly provided by Arun Srivastava, University of Florida, USA) was used, resulting in the hybrid serotype AAV1/mutAAV2 ^3^. The cells were harvested 48 h after transfection. Viral vectors were purified by dialysis and virus gradient centrifugation in iodixanol. For further enrichment, fast protein liquid chromatography was performed to obtain high titer viral stocks. Viral titers were determined by qPCR with appropriate plasmid standards. Toxicity and transduction rates of all AAV were tested in different dilutions in rat primary cortical neurons and after intravitreal injections *in vivo*.

**Microfluidic chambers**

The microfluidic chamber system consists of two main channels and four wells. The two main channels are connected by small microgrooves (7 µm wide, 3 µm high and 450 µm long). The chambers were fabricated based on previously published protocols ^4,5^. In brief, a 10:1 mixture of poly(dimethylsiloxane) (PDMS) prepolymer and cross-linker (Sylgard 184, Dow Corning) was poured onto a master mold (fabricated by photolithograpy) and cured for 90 min at 60°C. The cured PDMS piece was cut out, removed from the master mold and sterilized in 70% ethanol. It was then placed on a glass coverslip coated with 0.1 mg/mL PDL and used for cell culture.

**Western blot analysis**

For Western blots of optic nerves and retinas, rats were [sacrificed](http://www.google.de/search?biw=1350&bih=629&q=sacrificed&spell=1&sa=X&ei=O_juUYKZI8eRtAbPpYHYDA&ved=0CCcQBSgC) by CO_2_ insufflation. Optic nerves were dissected in the regions 1 mm proximal and distal to the crush site at the given time points after ONC. The samples were immediately frozen in liquid nitrogen, and then homogenized in lysis buffer (10 mM Hepes pH 7.2, 142 mM KCl, 5 mM MgCl_2_.6H_2_O, 1 mM EGTA, 1mM DTT, 1% IGEPAL, complete protease inhibitor, 50 µL phosSTOP-phosphatase inhibitor). For Western blot of cortical neurons, cells were lysed in the same lysis buffer. Protein lysates were sonicated and centrifuged. Protein concentration was measured by bicinchoninic acid (BCA) assay. Equal amounts (10-20 µg) of protein were loaded in each lane and separated by SDS-polyacrylamide gel electrophoresis (SDS-PAGE). Proteins were then transferred to a polyvinylidene difluoride (PVDF) or nitrocellulose (NC) membrane at 100 V for 2 h. After blocking the membrane for 1 h at room temperature (RT), they were incubated with primary antibodies at 4 °C overnight and then incubated with HRP-coupled secondary antibodies at RT for 1 h. The signal was visualized by ECL-reagent and quantified by ImageJ 1.49a software (NIH).

**RNA isolation and analysis**

Total RNA was isolated from AAV-treated cortical neurons on DIV8, or from optic nerves four weeks after intravitreal injection of AAVs. Cells or optic nerves were homogenized with Trizol reagent. 1-Bromo-3-chlor-propane was added to the lysates. After centrifugation for 15 min, isopropanol and glycoblue were added to the extraction of the upper aqueous layer. The mixture was kept at -20 °C overnight followed by centrifugation. Afterwards, the RNA pellet was washed with prechilled 75% ethanol. The dried pellet was dissolved in DEPC-H_2_O. The quantity of RNA was measured using a Nanodrop spectrophotometer (Thermo Scientific, Wilmington, DE, USA). The quantiTect reverse transcription kit (Qiagen) was used for reverse transcription. Real-Time quantitative PCR was conducted with QuantiTect SYBR green PCR kit (Qiagen) with a thermal cycler (Bio-Rad C1000 Touch). The primer of GAPDH was Rn_Gapd_1_SG (NM_017008) (QT00199633) (Qiagen). The sequences of human specific CRMP2 primers were: CRMP2 forward primer: 5’-CGTGAATCGTGCCATCACCA-3’; CRMP2 reverse primer: 5’-AGTAATGGGAGCCGTCCGTT-3’. The expression of CRMP2 mRNA was normalized to the mRNA expression of GAPDH according to the Bio-Rad CFX manager software (Bio-Rad).

**Proteomics analysis**

For mass spectrometric analysis, samples were enriched on a self-packed reversed phase-C18 precolumn (0.15 mm ID x 20 mm, Reprosil-Pur120 C18-AQ 5 µm, Dr. Maisch, Ammerbuch-Entringen, Germany) and separated on an analytical reversed phase-C18 column (0.075 mm ID x 250 mm, Reprosil-Pur 120 C18-AQ, 3 µm, Dr. Maisch) using a 30 min linear gradient of 5-35 % acetonitrile/0.1% formic acid at 300 nl/min). The eluent was analyzed on a TripleTOF 5600+ hybrid quadrupole/time-of-flight (QqTOF) mass spectrometer (AB SCIEX, Darmstadt) equipped with a nanoSpray III ion source and operated under Analyst TF1.6 software build 6211 using a data-dependent acquisition method. Each experimental cycle was of the following form: one full MS scan across the 350-1250 *m/z* range was acquired at a resolution of 30,000 FWHM and an accumulation time of 250 ms to select up to the 15 most abundant peptide precursors of charge states 2 to 4 above a 250 cps intensity threshold. Precursors were then isolated at 0.7 FWHM isolation width, fragmented with nitrogen at default rolling collision energy settings, and the resulting product ion spectra recorded across the 180-1600 *m/z* range at a resolution of 17,500 FWHM and an accumulation time of 100 ms. Selected precursor *m/z* values were then excluded for the following 9 s. Two technical replicates per sample were acquired.

For data processing, protein identification was achieved using ProteinPilot 5.0 software rev4769 (AB SCIEX, Darmstadt). Proteins were identified against the UniProtKB rat reference proteome v2015.02 (58766 protein entries) along with a set of 51 contaminants commonly identified in our laboratory. The search was performed at “thorough” search settings, with trypsin as enzyme and iodoacetamide as cysteine blocking agent. Results were exported into mzIdentML 1.1.0 format for further processing. Scaffold software version 4.4.1.1 (Proteome Software Inc., Portland, OR) was used to validate MS/MS based peptide and protein identifications. Peptide identifications were accepted if they could be established at greater than 95.0% probability by the Paragon algorithm ^6^. Protein identifications were accepted if they could be established at greater than 31.0% probability to achieve an FDR less than 1.0% and contained at least 2 identified peptides. Protein probabilities were assigned by the Protein Prophet algorithm ^7^. Proteins that contained similar peptides and could not be differentiated based on MS/MS analysis alone were grouped to satisfy the principles of parsimony. Proteins sharing significant peptide evidence were grouped into clusters. Proteins were annotated with GO terms from NCBI downloaded Feb 23, 2015 ^8^. Relative quantification of proteins in the samples was achieved by Analysis of Variance (ANOVA) of normalized Spectral Counts using a Benjamini-Hochberg-corrected p value of 0.05 to judge significance. To allow for the calculation of low abundance protein ratios, a minimum value of 3 spectral counts was introduced where necessary to avoid division by zero issues. For the proteins significantly regulated between groups, an over-representation analysis of was performed using GeneTrail ^9^. For this purpose, UniProt entry names were mapped to Entrez gene ids using the UniProt Retrieve/ID mapping tool. Fold changes between uninjured and injured optic nerves were calculated for the proteins in the pathways linked to axonal degeneration. The proteins with less than 5 spectral counts were excluded from analysis. Protein levels are considered altered when they have an expression ratio ≥1.3 or ≤ -1.3 compared to control.

**Animal experiments**

All animal experiments were carried out according to the regulations of the local animal research council and legislation of the State of Lower Saxony. Adult female Wistar rats (250–350 g, Charles River) were used in all experiments. Anesthesia was administered via [intraperitoneal injection](http://en.wikipedia.org/wiki/Intraperitoneal_injection) of 2% xylazine (7 mg/kg body weight) and 10% ketamine (95 mg/kg body weight).

**References**

1. Koch, J. C., Barski, E., Lingor, P., Bähr, M. & Michel, U. Plasmids containing NRSE/RE1 sites enhance neurite outgrowth of retinal ganglion cells via sequestration of REST independent of NRSE dsRNA expression. *FEBS J.* **278,** 3472–3483 (2011).

2. Rogemond, V. *et al.* Processing and nuclear localization of CRMP2 during brain development induce neurite outgrowth inhibition. *J. Biol. Chem.* **283,** 14751–14761 (2008).

3. Zhong, L. *et al.* Next generation of adeno-associated virus 2 vectors: point mutations in tyrosines lead to high-efficiency transduction at lower doses. *Proc. Natl. Acad. Sci. U. S. A.* **105,** 7827–7832 (2008).

4. Park, J. W., Vahidi, B., Taylor, A. M., Rhee, S. W. & Jeon, N. L. Microfluidic culture platform for neuroscience research. *Nat. Protoc.* **1,** 2128–2136 (2006).

5. Rhee, S. W. *et al.* Patterned cell culture inside microfluidic devices. *Lab Chip* **5,** 102–107 (2005).

6. Shilov, I. V *et al.* The Paragon Algorithm, a next generation search engine that uses sequence temperature values and feature probabilities to identify peptides from tandem mass spectra. *Mol. Cell. Proteomics* **6,** 1638–1655 (2007).

7. Nesvizhskii, A. I., Keller, A., Kolker, E. & Aebersold, R. A statistical model for identifying proteins by tandem mass spectrometry. *Anal. Chem.* **75,** 4646–4658 (2003).

8. Ashburner, M. *et al.* Gene ontology: tool for the unification of biology. The Gene Ontology Consortium. *Nat. Genet.* **25,** 25–29 (2000).

9. Backes, C. *et al.* GeneTrail-advanced gene set enrichment analysis. *Nucleic Acids Res.* **35,** 186–192 (2007).

**Supplementary figures**

**
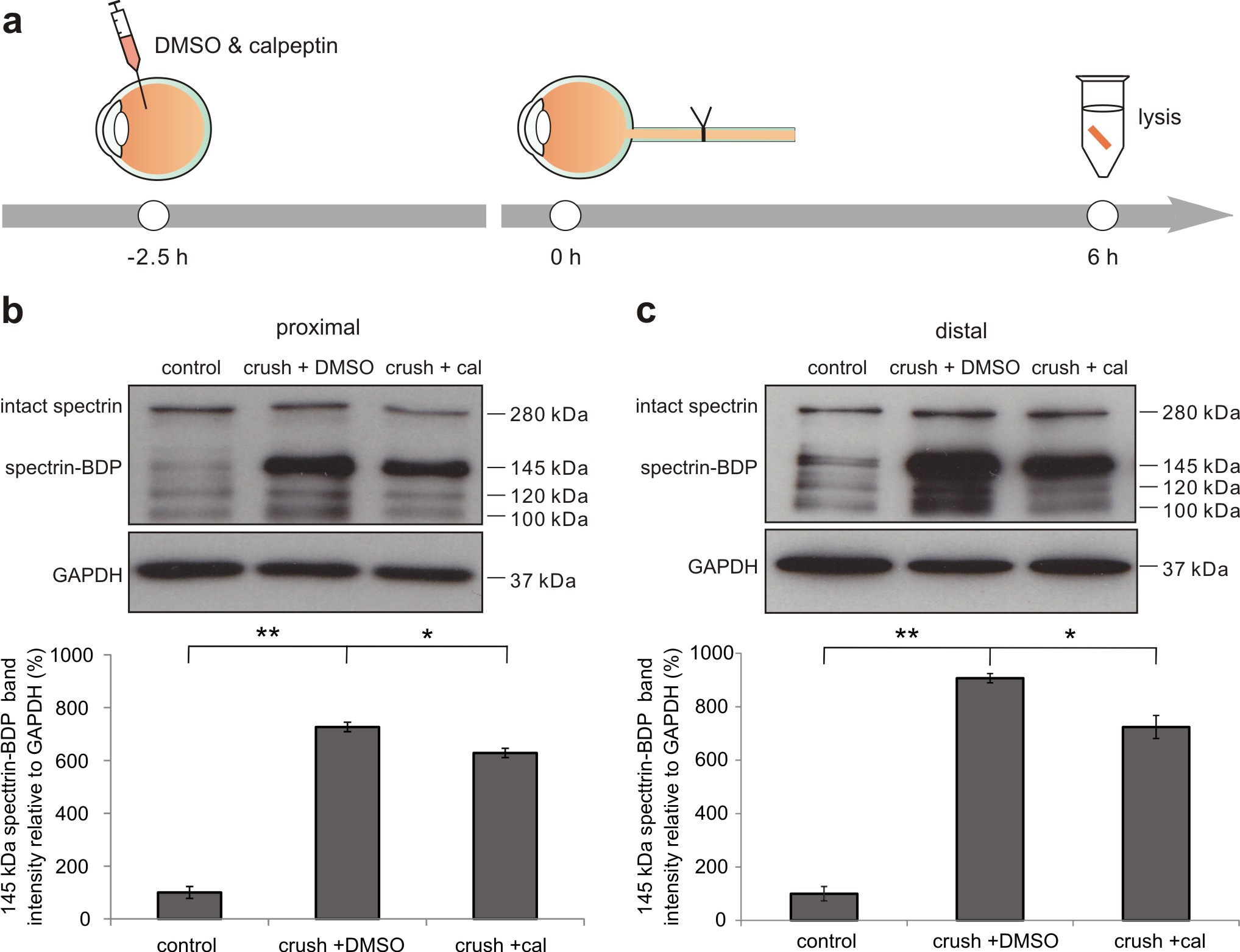
**

**Supplementary Figure S1.** Effect of calpeptin on calpain-mediated cleavage of spectrin after optic nerve crush *in vivo*

(a) Experimental setup. 10 mM calpain inhibitor calpeptin in 7% DMSO or 7% DMSO (3 µL each) was injected intravitreally 2.5 h before optic nerve crush. Segments of the optic nerve 1 mm proximal or distal to the lesion site were dissected, and lysates were made at 6 h after crush.

(b, c) Representative Western blots of spectrin proximal (b) and distal (c) to the crush site are shown. Cal = calpeptin. Experimental conditions include native optic nerves, optic nerves at 6 h after crush pretreated with 7% DMSO, and optic nerves at 6 h after crush pretreated with 10 Mm calpeptin in 7% DMSO (control, crush + DMSO & crush + cal). Below, the quantification of 145 kDa cleaved spectrin band intensity normalized to GAPDH in both proximal (b) and distal (c) parts to the crush site. 3 optic nerves per group. Error bars represent the standard error of the mean (SEM). **P* < 0.05, ***P* < 0.01 by one-way ANOVA and Dunnett’s test.

**
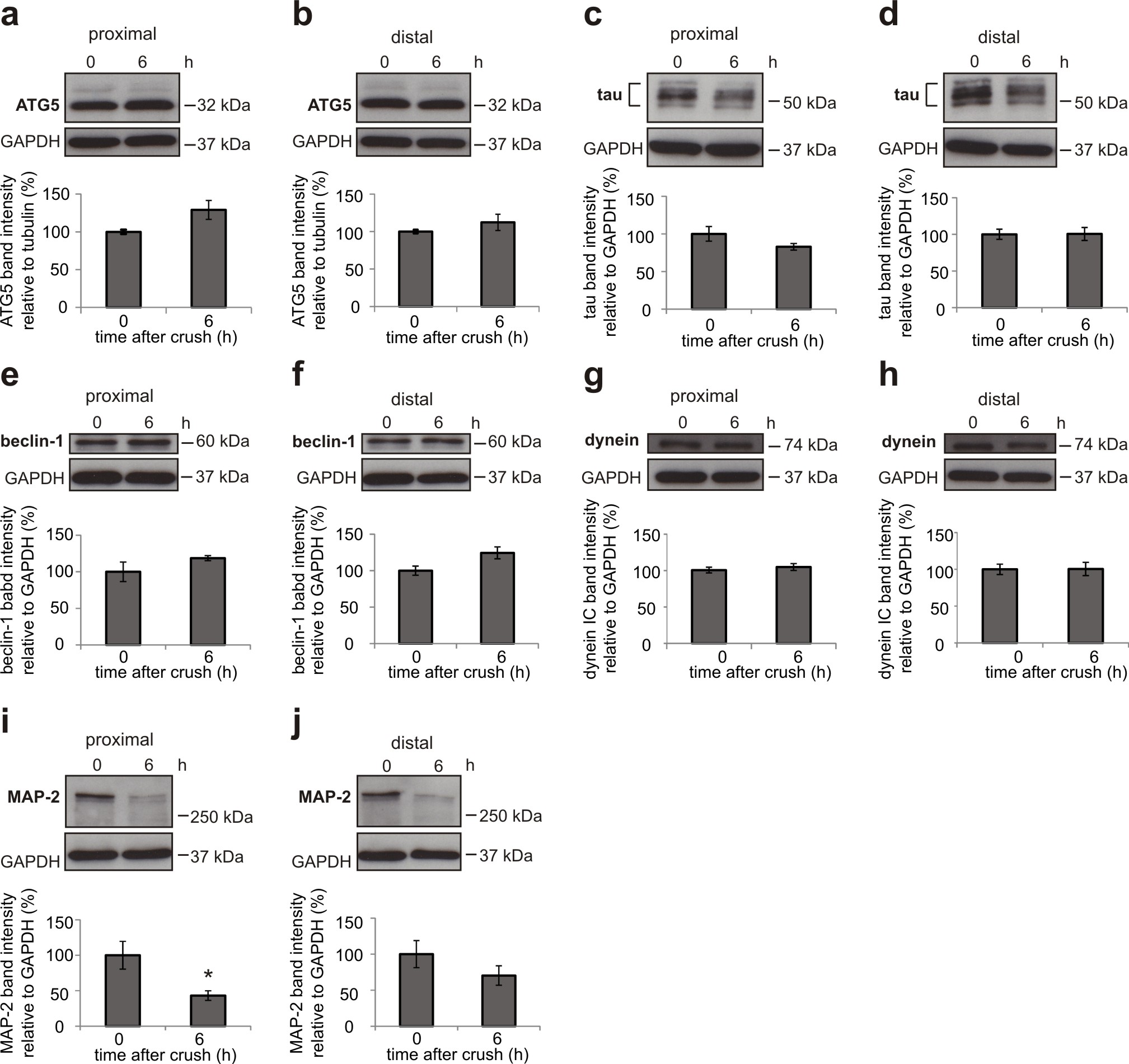
**

**Supplementary Figure S2.** Immunoblot analysis of putative targets for calpain-cleavage during acute axonal degeneration *in vivo*

(a-j) Representative Western blots of ATG5, tau, beclin-1, dynein IC and MAP-2 proximal and distal to the crush site in uncrushed native optic nerves (0 h) and in optic nerves at 6 h after crush are shown. Dynein IC = dynein intermediate chain. Below, the corresponding quantifications of ATG5, tau, beclin-1, dynein IC and MAP-2 band intensity relative to GAPDH are shown. 4 optic nerves per group. Error bars represent the standard error of the mean (SEM). **P* < 0.05 by independent samples t-test.

**
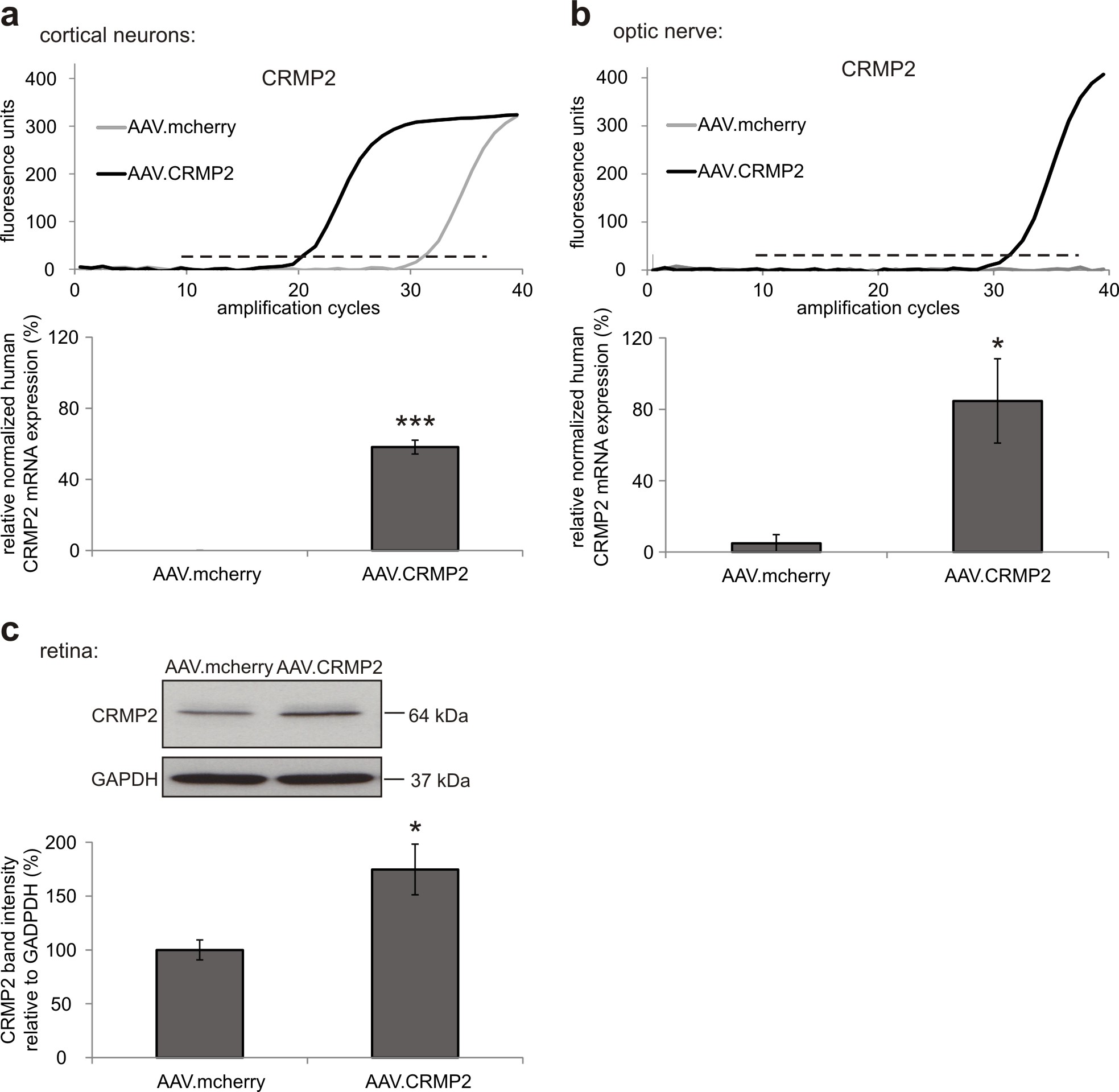
**

**Supplementary Figure S3.** Overexpression of CRMP2 by AAV.CRMP2 *in vitro* and *in vivo*

(a-b) Representative amplification results for human CRMP2 primer assessed SYBR green fluorescence intensities (y axis) per PCR cycle (x axis) in cortical neurons (a) and optic nerves (b) are shown. At the bottom, relative human CRMP2 mRNA expression levels were quantified and normalized to GAPDH mRNA expression. In (a), 3 independent cultures are included. In (b), 3 optic nerves are included in each group. Error bars represent the standard error of the mean (SEM). ****P* < 0.001 by independent samples t-test.

(c) Representative Western blot of CRMP2 in the retinal 4 weeks after transduction of AAV.mcherry or AAV.CRMP2 is shown. Below, quantification of the CRMP2 band intensity normalized to GAPDH. 3 retinas per group. Error bars represent the standard error of the mean (SEM). **P* < 0.05 by independent samples t-test.


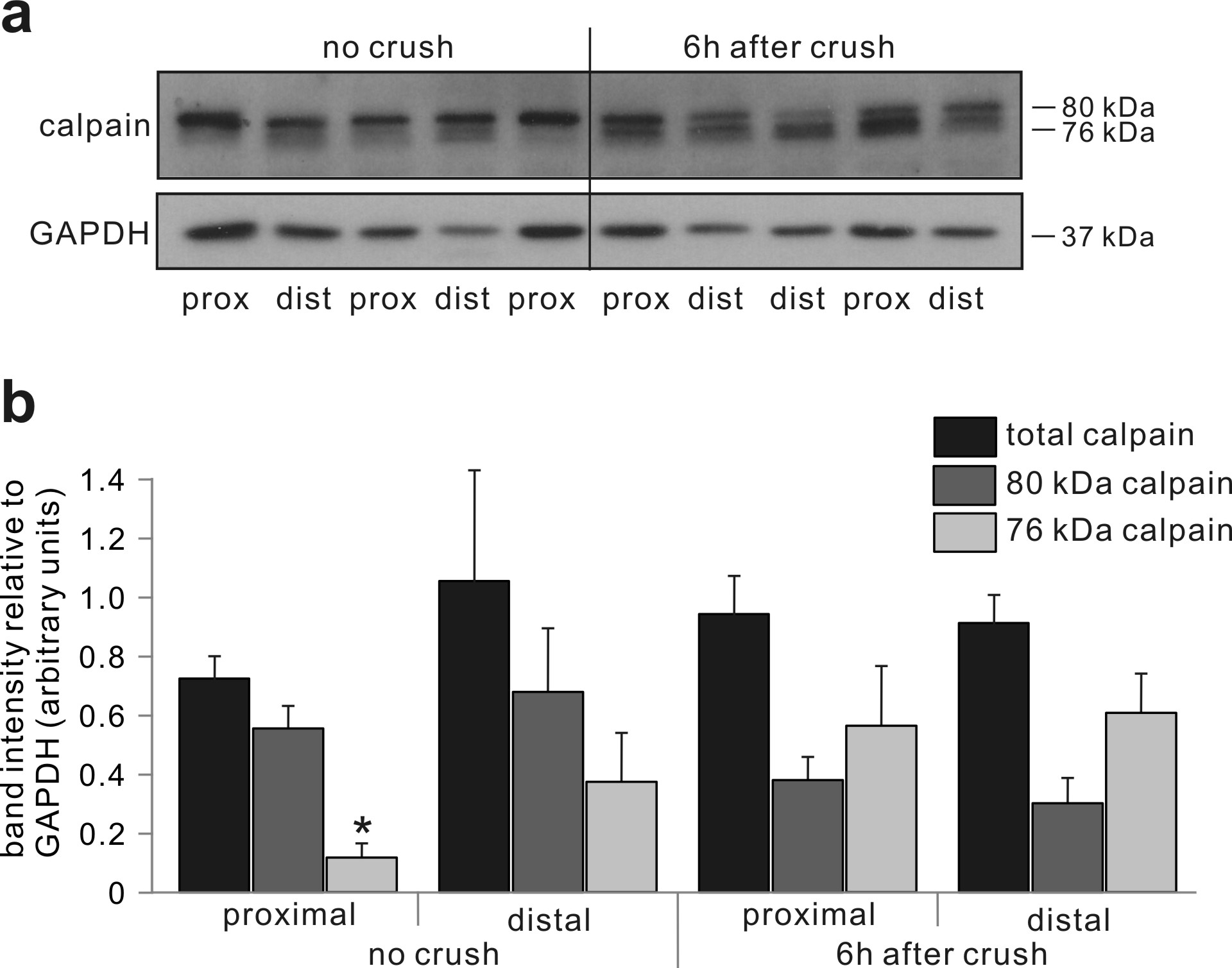


**Supplementary Figure S4:** Calpain levels after optic nerve crush.

(a) Western Blot of optic nerve lysates from proximal (prox) and distal (dist) parts of 3 independent rat optic nerves without lesion or 6h after crush lesion, respectively (1 probe unlesioned distal and 1 probe 6h after crush proximal were lost during the procedures, thus only 10 samples in total could be evaluated) (“proximal” and “distal” refer to the optic nerve tissue comprising 1 mm on both sides of the crush). Two isoforms of calpain can be differentiated at 80 and 76 kDa. The latter has been described as the proteolytical active form of calpain.

(b) Quantification of the band intensities of the Western Bot in (a). Total calpain refers to the quantification of both calpain bands together; there were no significant differences between the groups. The 80 kDa calpain band was decreased by trend at 6h after crush compared to the unlesioned optic nerve. In contrast, the 76 kDa band was increased significantly at 6h after crush on the proximal side and increased by trend on the distal side. 3 optic nerves per group. Error bars represent the standard error of the mean (SEM). *P < 0.05 by one-way ANOVA and Tukey’s test.

**Supplementary Table S1.** List of proteins linked to CRMP2 with significantly altered expression during acute axonal degeneration

| uniProtID | protein | function | ratio (proximal) | ratio (distal) |
| --- | --- | --- | --- | --- |
| P04636 | MDH2 | mitochondrial protein | 2.0 | 1.8 |
| P16086 | SPTAN1 | cytoskeletal protein | 2.3 | 1.4 |
| G3V6S0 | SPTBN1 | cytoskeletal protein | 3.1 | 1.9 |
| Q9QXQ0 | ACTN4 | regulation of actin cytoskeleton | 1.9 | -1.1 |
| Q91Y81 | SEPT2 | regulation of actin and tubulin | -1.1 | 1.5 |
| Q8CFN2 | CDC42 | regulation of actin and tubulin | -1.1 | 2.2 |
| P62260 | YWHAE | regulation of intermediate filaments | -1.4 | 1.5 |
| P54311 | GNB1 | signal transduction | -1.6 | 1.0 |
| P13221 | GOT1 | glutamate metabolism | -1.6 | -1.1 |
| Q5U318 | PEA-15 | activation of the ERK/MAPK pathway | -3.3 | -2.1 |
| P54311 | GNB1 | signal transduction | -1.6 | 1.0 |
| Q497B0 | NIT2 | glutamate and asparagine metabolism | -1.7 | -1.3 |
| F1M9C8 | KIF1C | motor protein of axonal transport | -3.7 | -3.7 |

The given proteins were identified by a STRING (v10 for rat) database search as previously reported interacting partners of CRMP2. Their expression levels are significantly altered at 6 h after ONC on the proximal and/or distal site of the lesion as compared to control without lesion. The respective fold-changes are given in the columns on the right side. Statistical analysis was performed using ANOVA and a Benjamini-Hochberg-corrected p value of 0.05 to judge significance. Each value was derived from four pooled optic nerves and was calculated as the average of two technical replicates. Protein levels are considered altered when they have an expression ratio ≥1.3 or ≤ -1.3 compared to control.
